# Report of the First International Consensus on Standardized Nomenclature of Antinuclear Antibody HEp-2 Cell Patterns 2014–2015

**DOI:** 10.3389/fimmu.2015.00412

**Published:** 2015-08-20

**Authors:** Edward K. L. Chan, Jan Damoiseaux, Orlando Gabriel Carballo, Karsten Conrad, Wilson de Melo Cruvinel, Paulo Luiz Carvalho Francescantonio, Marvin J. Fritzler, Ignacio Garcia-De La Torre, Manfred Herold, Tsuneyo Mimori, Minoru Satoh, Carlos A. von Mühlen, Luis E. C. Andrade

**Affiliations:** ^1^Department of Oral Biology, University of Florida, Gainesville, FL, USA; ^2^Central Diagnostic Laboratory, Maastricht University Medical Center, Maastricht, Netherlands; ^3^Laboratory of Immunology, Hospital Carlos G. Durand, Buenos Aires, Argentina; ^4^Department of Immunology, Instituto Universitario del Hospital Italiano, Buenos Aires, Argentina; ^5^Institute of Immunology, Technical University of Dresden, Dresden, Germany; ^6^Pontifícia Universidade Católica de Goiás, Goiânia, Brazil; ^7^Department of Medicine, Cumming School of Medicine, University of Calgary, Calgary, AB, Canada; ^8^Department of Immunology and Rheumatology, Hospital General de Occidente, University of Guadalajara, Guadalajara, Mexico; ^9^Department of Internal Medicine VI, Medical University of Innsbruck, Innsbruck, Austria; ^10^Department of the Control for Rheumatic Diseases, Graduate School of Medicine, Kyoto University, Kyoto, Japan; ^11^Department of Rheumatology and Clinical Immunology, Graduate School of Medicine, Kyoto University, Kyoto, Japan; ^12^Department of Clinical Nursing, University of Occupational and Environmental Health, Kitakyushu, Japan; ^13^Brazilian Society of Autoimmunity, Porto Alegre, Brazil; ^14^Rheumatology Division, Escola Paulista de Medicina, Universidade Federal de São Paulo, São Paulo, Brazil; ^15^Immunology Division, Fleury Medicine and Health Laboratories, São Paulo, Brazil

**Keywords:** antinuclear antibodies, autoantibody, autoimmunity, consensus, standardization

## Abstract

During the 12th International Workshop on Autoantibodies and Autoimmunity held in Sao Paulo, Brazil, on August 28, 2014, a full day session was devoted to establishing a consensus on the nomenclature of staining patterns observed in the antinuclear antibody (ANA) indirect immunofluorescence test on HEp-2 cells. The current report summarizes the collective agreements with input from the host Brazilian and international communities that represented research, clinical, and diagnostic service laboratories. Patterns are categorized in three major groups (nuclear, cytoplasmic, and mitotic patterns) and each pattern has been defined and described in detail. The consensus nomenclature and representative patterns are made available online at the international consensus on antinuclear antibody pattern (ICAP) website (www.ANApatterns.org). To facilitate continuous improvement and input, specific comments on ICAP are encouraged and these will be discussed in subsequent ICAP meetings. The ultimate goal with the establishment of the ICAP is to promote harmonization and understanding of autoantibody test nomenclature, as well as interpretation guidelines for ANA testing, thereby optimizing usage in patient care.

## Introduction

The antinuclear antibody (ANA) assay is commonly used as a laboratory indicator for the body’s autoimmune response (1, 2). Since the 1970s, HEp-2 cells have been increasingly used and were eventually adopted as the universal standard substrate in practically all commercially available ANA assay kits. There have been many continuing efforts to standardize ANA tests and one significant accomplishment was the development of autoantibody reference reagents (3). Over the past 25 years, the Autoantibody Standardization Committee, a subcommittee of the International Union of Immunological Societies (IUIS) Quality Assessment and Standardization Committee, has collaborated with the Centers for Disease Control and Prevention (CDC) and other agencies to provide autoantibody reference standards (also known as CDC ANA reference standards, or IUIS ANA reference standards). To date, there are 17 reference standards available free of charge to all qualified clinical or commercial laboratories and research investigators. The overall goal is to promote laboratory quality control of ANA and related autoantibody testing (4). However, the lack of inter-laboratory standardization and other problems in ANA testing and reporting persist, leading to recent discussions on the appropriate use of the ANA assay (5–7).

During the 12th International Workshop on Autoantibodies and Autoimmunity (IWAA) held in São Paulo, Brazil, a full day session attended by 66 experts was devoted to establishing an International Consensus on ANA staining Patterns (ICAP). Brazil has a long history of ANA pattern consensus with their first workshop held in Goiania in August, 2000. Their first consensus report was published in 2001 (8) and two subsequent ANA consensus reports published in 2003 (9) and 2009 (10). These earlier publications were in Portuguese with some including English abstracts. The report for the fourth Brazilian ANA consensus meeting in 2013 was published entirely in English (11). Other reports with a focus on nomenclature/consensus in ANA on HEp-2 cells are acknowledged and were referred to during the ICAP meeting and writing of this report (12–15).

This report summarizes the ICAP working consensus and this is also available online at the IWAA 2014 website with a link to a permanent website: www.ANApatterns.org. The ultimate goal with the implementation of ICAP is to promote harmonization of autoantibody test nomenclature and interpretation, and to maximize usages in patient care.

## Methods in Establishing Consensus

The critical need for the ICAP initiative was discussed and conceived during the planning stages of the IWAA meeting to be held in São Paulo. A full day agenda was designed for the ICAP initiative based on four initial discussion sessions that would focus on nuclear, nucleolar, cytoplasmic, and mitotic and complex patterns, respectively. This was followed by general discussion toward building a consensus and presentation of the findings to the wider IWAA conference forum. Two discussion leaders were selected for each session based in part on their expertise. Nuclear patterns were coordinated by Karsten Conrad (Germany) and Jan Damoiseaux (Netherlands). Nucleolar patterns were coordinated by Minoru Satoh (Japan) and Orlando Gabriel Carballo (Argentina). Cytoplasmic patterns were coordinated by Edward K. L. Chan (USA) and Carlos A. von Mühlen (Brazil). Mitotic and complex patterns were coordinated by Tsuneyo Mimori (Japan) and Manfred Herold (Austria). An additional 1-h session was devoted to technical recommendations presented by Ignacio Garcia-De La Torre (Mexico) and Paulo Luiz Carvalho Francescantonio (Brazil).

Prior to the IWAA meeting, information regarding the relevant literature on ANA consensus was provided to all discussion leaders. Each pair of coordinators was tasked to lead the assigned topic for discussion. This included collecting and organizing relevant ANA patterns into a proposed preliminary consensus. During the ICAP sessions, 66 registrants (Table S1 in Supplementary Material) representing 15 countries (Table S2 in Supplementary Material) attended the presentations by the discussion leaders and about two-thirds participated in the concluding question and answer sessions. Briefly, one or both of the discussion leaders presented an outline of the logic model classification tree that would be useful to differentiate staining patterns. Examples of each staining pattern were displayed and opened for discussion, and patterns that attained initial consensus were established and separated from others in need of further discussion. At the end of the day, an additional discussion session took place to plan for a final report to be presented as the summary of the first ICAP. It was agreed that deliberations on the major technical considerations of ANA immunoassays would be summarized in a separate report to follow the current manuscript. In addition, it was determined that the present report needed to be in place in the following months to ensure that a summation of the ICAP deliberations would be made available in a timely manner. Finally, it was established that a website would be constructed under the leadership of Wilson de Melo Cruvinel and Edward K. L. Chan with input and consultation with all participants as needed. The website would display the reference images for ongoing discussion by the leaders, thereby consolidating the consensus on the ANA patterns and nomenclature. In due time, the website will display the classification tree of the ANA patterns and representative images of each pattern. The primary goal of the website is to provide free access to the consensus patterns by the international community. There was agreement that ongoing efforts would be made to further improve guidelines by means of successive rounds of ICAP. The second ICAP meeting has been scheduled to take place on September 22, 2015, immediately prior to the 12th Dresden Symposium on Autoantibodies in Germany.

## Classification Tree

**Figure 1:**
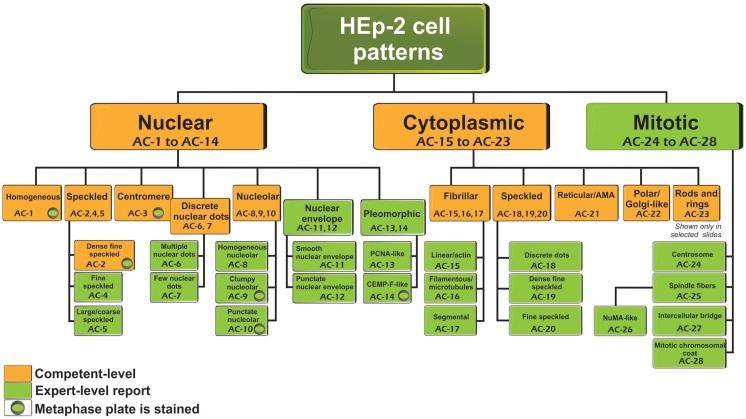
**Nomenclature and classification tree for nuclear, nucleolar, cytoplasmic, and mitotic IIF staining patterns on HEp-2 cell substrates**. This is a summary of the International Consensus on Antinuclear antibody Pattern (ICAP) meeting and subsequent discussion, debate, and dialog. Patterns are shown from AC-1 to AC-28. Examples of some of the major patterns are shown in Figures 2 and 3, while additional images of each are depicted in a web page linked to the ANA ICAP website (www.ANApatterns.org). Boxes with amber background are recommended as competent-level reporting, whereas those with olive green background are considered for expert-level reporting. AC, anti-cell.

Antinuclear antibody is the common clinical and laboratory term used for more than 50 years. However, the name “antinuclear” for the ANA test does not take into consideration that autoantibodies to cell compartments other than the nucleus are also detected. Nevertheless, the ANA term is maintained for historical reasons and also for laboratory coding and invoicing. Thus, in situations when there is clear cytoplasmic or mitotic apparatus reactivity, the ANA indirect immunofluorescence (IIF) test is to be reported as positive. The classification tree for most staining patterns is presented in Figure 1 and they are segregated into nuclear, cytoplasmic, and mitotic patterns. In accord with the Brazilian Consensus strategy (11), certain patterns are recommended for mandatory reporting, while others are for expert-level reporting. During the write-up of this manuscript, in addition to comments from the reviewers, there were some concerns that “mandatory” might not fit well with dictating the requirements for clinical immunology laboratories, especially in an international setting. The ICAP intention is to indicate patterns that should be readily recognized (competent-level) versus patterns that would be more challenging and distinguishable only when observers or technologists have attained the expert-level. The distinction between competent-level versus expert-level patterns is based on at least two considerations. First, clinical relevance probably is a major consideration to ensure that important clinical implications are recognized. Second, easily recognizable patterns should be included even when the clinical relevance is less clear at this time. It is acknowledged that the current separation of competent-level patterns (amber boxes) and expert-level patterns (olive green boxes) is a temporary status that may change over time. However, competent-level patterns are strongly recommended for reporting. The competent-level patterns are placed at the top levels starting from the left. Each pattern is assigned a code below the descriptor. For example, the nuclear homogeneous box has the code *a*nti-*c*ell pattern 1 (AC-1). These codes allow for easy and objective access and reference to the web-based consensus patterns available on the ICAP website (www.ANApatterns.org). The assignment of the different AC codes generally flows from left to right, and top to bottom. Thus, the classification tree shows 11 competent-level reportable patterns. The six competent-level reportable nuclear patterns include homogeneous, speckled, dense fine speckled, centromere, discrete nuclear dots, and nucleolar. Five competent-level reportable cytoplasmic patterns are fibrillar, speckled, reticular/mitochondrion-like, polar/Golgi-like, and rods and rings (RR). The RR pattern is not recognized in certain commercial ANA substrates as these structures are only seen consistently in slides from some manufacturers (16–18). It is acknowledged that not all known ANA patterns are shown in Figure 1 and that new editions of the ICAP may add new patterns to this consensus classification tree. For example, mixed patterns that may originate from a mixture of one or more simple patterns are commonly observed but it was decided that they would not be included in the first iteration of this exercise. One point that did not reach consensus was the proposal to develop a separate category of “composite patterns,” such as those in which single autoantibody specificity yields a combination of staining of different cell compartments (e.g., NuMA, topoisomerase I).

## Nuclear Patterns

**Figure 2:**
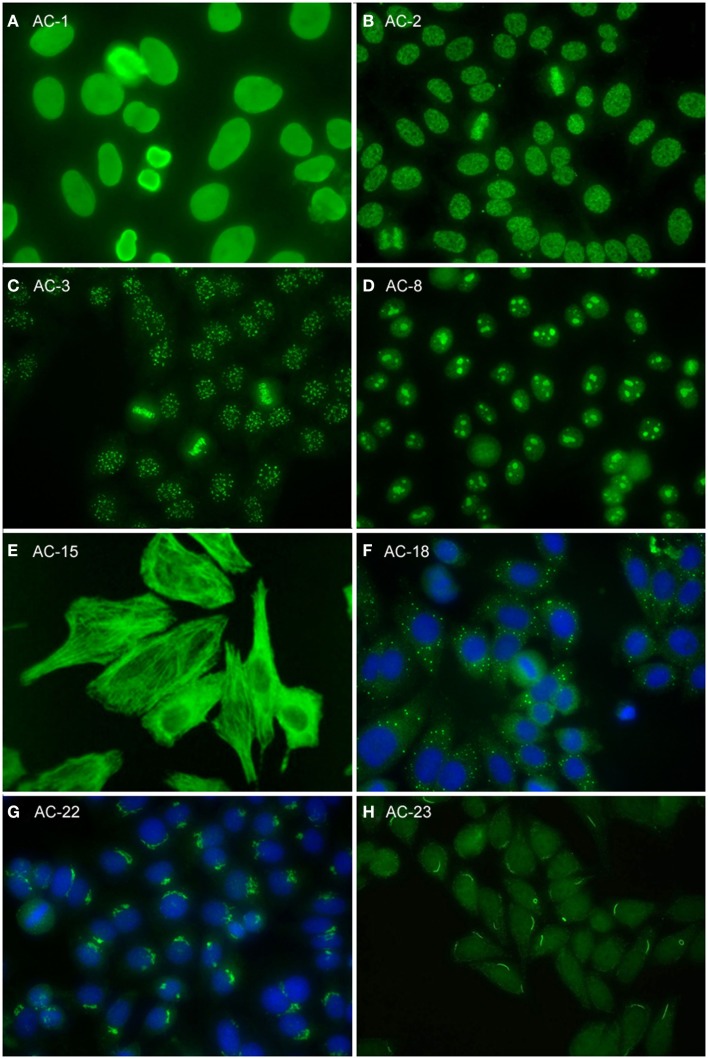
**Representative images of selected major HEp-2 cell patterns**. **(A)** homogeneous nuclear (AC-1); **(B)** nuclear dense fine speckled (AC-2); **(C)** centromere (AC-3); **(D)** homogeneous nucleolar (AC-8); **(E)** cytoplasmic fibrillar linear (AC-15); **(F)** cytoplasmic discrete dots (AC-18); **(G)** polar/Golgi-like (AC-22); **(H)** rods and rings (AC-23).

Nuclear patterns are defined as any staining of the HEp-2 interphase nuclei, irrespective of positive or negative staining of mitotic cells. In total, 6 major pattern groups (top row under nuclear patterns, Figure 1) and 11 minor pattern subgroups can be recognized based on the staining of distinct nuclear compartments in interphase cells (Table 1). Representative images of the major patterns are shown in Figure 2. The respective nomenclature is primarily based on the reactivity observed in the nucleoplasm (cf. *homogeneous* or *speckled*) and the nuclear subcomponents that are recognized (cf. *centromere* or *nucleolar*). Homogeneous, Speckled, Centromere, and Nucleolar are major pattern groups considered as competent-level report for all laboratories that perform ANA IIF tests. If there is a very strong correlation of the IIF pattern with the target autoantigen that is recognized, the common name of the respective antigen is used with addition of “-like” (cf. PCNA-like). It should be noted that the use of the term “-like” to describe patterns is that the observation of the pattern itself should rarely be taken as the ultimate determination of the autoantibody specificity. Instead, further characterization is recommended including co-localization with known marker antibodies, Western blot, immunoprecipitation, double immunodiffusion, ELISA, dot or line blots, chemiluminescence, or other solid phase immunoassays. Synonyms based on historical nomenclature of the distinct patterns are listed in Table 2. This table also includes associations of the distinct patterns with the autoantigen specificity (when known) as well as diseases.

**Table 1:** **Nuclear patterns defined by reactivity with distinct nuclear compartments in interphase cells and staining of mitotic cells**.

	Nucleoplasm	Nucleoli	Metaphase chromosomal plate	Metaphase cytoplasm	Mitotic apparatus	Interphase cytoplasm
**Homogeneous** (AC-1)	Homogeneous	Negative/positive	Homogeneous	Negative	Negative	Negative

**Speckled**						
**Dense fine speckled** (AC-2)	Heterogeneous fine speckles	Negative	Heterogeneous fine speckles	Negative	Negative	Negative
Fine speckled (AC-4)	Uniform fine speckles	Negative/positive	Negative	Diffuse fine speckles	Negative	Negative
Large/coarse speckled (AC-5)	Variably sized large speckles	Negative	Negative	Diffuse fine speckles	Negative	Negative

**Discrete nuclear dots**						
Centromere (AC-3)	~30–50 dots	Negative	~30–50 aligned dots	Negative	Negative	Negative
Multiple nuclear dots (AC-6)	~10 dots	Negative	Rarely occasional dots	Negative/positive	Negative	Negative
Few nuclear dots (AC-7)	1–6 dots	Negative	Rarely occasional dots	Negative	Negative	Negative

**Nucleolar**						
Homogeneous (AC-8)	Negative	Homogeneous	Negative	Diffuse homogeneous	Negative	Negative
Clumpy (AC-9)	Negative	Large granular	Positive (peri-chromosomal)	Negative/positive	Negative	Negative
Punctate (AC-10)	Negative	Fine speckled	1–5 bright pairs of spots (NOR)	Negative/positive	Negative	Negative

**Nuclear envelope**						
Smooth NE (AC-11)	Linear staining of NE	Negative	Negative	Diffuse	Negative	Negative
Punctate NE (AC-12)	Granular staining of NE	Negative	Negative	Homogeneous/dense speckled	Negative	Negative

**Pleomorphic**						
PCNA-like (AC-13)	Variably sized speckles in S-phase cells (~30%)	Positive in late S-phase	Negative	Negative	Negative	Negative
CENP-F-like (AC-14)	Fine granular in G2-phase	Negative	~30–50 aligned dots	Diffuse	Midbody	Negative

*Amber background are recommended as competent-level reporting, whereas all others (Olive green) are considered for expert-level reporting*.

**Table 2:** **Synonyms for nuclear patterns and association with specific antigens and diseases**.

		Synonyms	Antigen associations	Disease association
*Nuclear patterns*
	**Homogeneous** (AC-1)	Diffuse	dsDNA, nucleosomes, histones	SLE, drug-induced lupus, juvenile idiopathic arthritis

	**Speckled** (AC-2,4,5)	Granular	hnRNP, U1RNP, Sm, SS-A/Ro (Ro60), SS-B/La, RNA polymerase III, Mi-2, Ku	MCTD, SLE, SjS, DM, SSc/PM overlap
	***Dense fine speckled*** (AC-2)	None	DFS70/LEDGF	Rare in SLE, SjS, SSc
	Fine speckled (AC-4)	Fine granular	SS-A/Ro (Ro60), SS-B/La, Mi-2, TIF1γ, TIF1β, Ku, RNA helicase A, Replication protein A	SjS, SLE, DM, SSc/PM overlap
	Large/coarse speckled (AC-5)	Spliceosome/nuclear matrix	hnRNP, U1RNP, Sm, RNA polymerase III	MCTD, SLE, SSc

	**Discrete nuclear dots**			
	Centromere (AC-3)	Kinetochore	CENP-A/B (C)	Limited cutaneous SSc, PBC
	Multiple nuclear dots (AC-6)	6–20 nuclear dots, NSpI, PML bodies	Sp100, PML proteins, MJ/NXP-2	PBC, SARD, PM/DM
	Few nuclear dots (AC-7)	1–6 nuclear dots, Cajal bodies (coiled body)	p80-coilin, SMN	SjS, SLE, SSc, PM, asymptomatic individuals

	**Nucleolar** (AC-8,9,10)			
	Homogeneous (AC-8)	None	PM/Scl-75, PM/Scl-100, Th/To, B23/nucleophosmin, nucleolin, No55/SC65	SSc, SSc/PM overlap
	Clumpy (AC-9)	None	U3-snoRNP/fibrillarin	SSc
	Punctate (AC-10)	Nucleolar speckled	RNA polymerase I, hUBF/NOR-90	SSc, SjS

	**Nuclear envelope** (AC-11,12)			
	Smooth nuclear envelope (AC-11)	Nuclear rim, nuclear membrane, membranous	Lamins A,B,C, or lamin-associated proteins	SLE, SjS, seronegative arthritis
	Punctate nuclear envelope (AC-12)	Nuclear membrane pores	Nuclear pore complex proteins (i.e., gp22)	PBC

	**Pleomorphic** (AC-13,14)			
	PCNA-like (AC-13)	None	PCNA	SLE, other conditions
	CENP-F-like (AC-14)	MSA-3, NSp-II	CENP-F	Cancer, other conditions

*These disease associations are primarily based on the target antigens recognized by autoantibodies that reveal a particular ANA pattern. Amber background are recommended as competent-level reporting, whereas all others (Olive green) are considered for expert-level reporting*.

### Homogeneous

The homogeneous nucleoplasmic staining is the first major group of nuclear patterns that is considered competent-level in terms of recognition and reporting. It is characterized by a diffuse and uniform staining (Figure 2A). The nucleolar region usually is also stained, but occasionally may not show any staining. High titer sera may show more pronounced staining at the outer rim of interphase nuclei. The chromatin plate of metaphase cells also shows homogeneous staining, with a clearly hyaline, or diffuse, appearance. The cytoplasm is typically negative in interphase and mitotic cells. The homogeneous pattern is associated with antibodies directed to chromatin components, such as dsDNA, core histones, and/or nucleosomes. It is of utmost importance that the homogeneous pattern should be differentiated from the dense fine speckled (DFS) pattern in routine practice since the clinical significance of both patterns is quite different (see next paragraph).

### Speckled

The speckled nuclear pattern is a major group that is to be recognized and reported by all clinical laboratories. The distinction of a fine or large/coarse speckled pattern, based on the size of the speckles in interphase cells, is only recommended for expert-level laboratories. The distinctive feature of the speckled pattern is the granular staining of the nucleoplasm of interphase cells. The large/coarse speckled pattern is characterized by dense intermediate sized speckles in the nucleus associated with larger speckles throughout the nucleoplasm of interphase cells. Typically, both nucleoli and mitotic chromatin show no staining. The fine speckled pattern shows a fine granular, sometimes very dense, staining of the nucleus in a uniform distribution. Nucleoli may stain (e.g., SS-B/La or Ku antibodies) or are negative. The chromatin plate is usually negative with some exceptions, like DNA topoisomerase I antibodies. The cytoplasm of metaphase cells of both large and fine speckled nuclear patterns reveals a speckled pattern that may be more condensed around the chromatin plate; however, this characteristic is not critical in defining this pattern. A key challenge, though, is to distinguish the DFS pattern from both the homogeneous and the speckled pattern. The DFS pattern is characterized by a unique dense and heterogeneous speckled staining of both the nucleoplasm of interphase cells and, in contrast with most other speckled patterns, the metaphase chromosomal plate (Figure 2B). One distinctive peculiarity of this speckled pattern is heterogeneity of the size, brightness, and density of the speckles throughout the interphase nucleoplasm (19, 20). This composite pattern is considered clinically relevant since it indicates that the presence of SLE, SjS, or SSc is unlikely (19, 21–24). Therefore, the DFS pattern is classified for competent-level reporting.

### Centromere and discrete nuclear dots

The centromere pattern is considered a subset of the discrete nuclear dots patterns (Figure 2C). This most characteristic pattern is to be recognized and reported by all clinical laboratories and, therefore, placed at the same level as homogeneous and speckled. In the classification tree, the discrete nuclear dots box is overlapping with centromere to symbolize their relationship – that they are both discrete nuclear dots and yet readily distinguishable and with different clinical associations. The centromere pattern shows multiple, somewhat uniform, discrete dots distributed throughout the entire nucleus. Mitotic cells also exhibit this speckled/dot pattern in a typical alignment within the condensed chromosomal material. The nucleoli are usually negative, although sometimes some dots may cluster within the nucleolar area. Antibodies revealing this pattern react with proteins localized to the kinetochores of chromosomes.

The remaining discrete nuclear dot patterns are subdivided based on the number and localization of the dots in the nuclei of interphase cells. The multiple nuclear dot pattern characteristically reveals on average 10 discrete dots (often range from 6 to 20 dots), variable in size, in the nucleus of interphase cells. The chromosomal plates of mitotic cells do not typically stain, but cytoplasmic staining may be observed. These nuclear dots are known as promyelocytic leukemia (PML) nuclear bodies (25, 26). Some of the associated antigens include PML proteins, such as Sp100 (26), and the more recently described MJ/NXP-2 antigen (27). The few nuclear dots pattern shows only 1–6 dots per nucleus, often in close proximity to nucleoli. Cells with higher number of dots (4–6) are in the late S/G2 phase of the cell cycle (28); metaphase chromatin in mitotic cells is usually negative. These nuclear dots are Cajal bodies (formerly known as coiled bodies) and the primary antigen is p80-coilin (29) and survival of motor neuron (SMN) (30).

### Nucleolar

Nuclear staining that is predominantly restricted to the nucleoli of interphase cells is referred to as the nucleolar pattern. This pattern is considered as competent-level reporting for clinical laboratories. Based on the staining pattern within the nucleoli, three subtypes may be distinguished in expert-level laboratories. The homogeneous nucleolar pattern is characterized as diffuse staining of the entire nucleolus and may present a relatively much weaker diffuse staining of the nucleoplasm, while the cytoplasm of mitotic cells tends to be diffusely stained (Figure 2D). Irregular staining of the nucleoli, revealing clustered large granules, is referred to as clumpy nucleolar pattern. The classical autoantibody specificity associated with this clumpy nucleolar pattern is anti-U3 RNP/fibrillarin, which is also known to stain Cajal bodies (31). In mitotic cells, the outer surface of the condensed chromatin is often stained, while the cytoplasm of mitotic cells may be weakly positive. The punctate nucleolar pattern is characterized by densely distributed but distinct dots in the nucleoli of interphase cells. In metaphase cells, up to five bright pairs of the nucleolar organizer regions (NOR) can be seen as bright discrete dots within the chromatin mass. The cytoplasm of mitotic cells may show weak diffuse staining.

### Nuclear envelope

Staining of the nuclear envelope (NE) may reveal either a continuous linear or punctate staining of interphase cells, i.e., smooth NE or punctate NE. In metaphase cells, the fluorescence is diffusely localized throughout the cytoplasm, leaving the metaphase plate unstained. Although both the major nuclear category and both subgroups are considered expert-level reporting for all clinical laboratories, this reactivity is considered to be a definable ANA pattern with a limited repertoire of autoantibody specificities and their respective clinical significance.

### Pleomorphic

Both the proliferating cell nuclear antigen (PCNA) pattern and the CENP-F pattern are considered subtypes of the pleomorphic nuclear staining category, because they show variable staining characteristics at distinct stages of the cell cycle. Neither the general pleomorphic pattern nor the subtypes are to be reported by all clinical laboratories. However, these patterns are to be reported as nuclear pattern ANA positive.

The PCNA-like staining pattern is characterized by variable sized speckles in the nucleoplasm, which achieves maximum intensity and density of speckles in the S-phase of cell cycle. Depending on the HEp-2 cell growth characteristics and slide preparation, this is represented in about 30% of the HEp-2 cells. In late S-phase and early G2 phase, the speckles become progressively sparser and the nucleoli are also stained. G1 cells and metaphase cells typically do not stain.

On the other hand, the CENP-F-like pattern shows fine granular staining of the nucleoplasm, but not the nucleoli of interphase cells. There is striking variability in intensity with the strongest staining in G2 phase and weakest/negative staining in G1. Centromere staining is seen only in prometaphase and metaphase cells, revealing multiple aligned small and faint dots. Prometaphase cells frequently show a weak staining of the nuclear envelope. The surrounding cytoplasm of the mitotic cells is typically diffusely stained. While some anti-CENP-F sera also show staining of the midbody, this is not a constant feature associated with anti-CENP-F.

Proliferating cell nuclear antigen and CENP-F staining patterns are labeled as PCNA-like and CENP-F-like for the obvious reason that their staining patterns are highly characteristic for autoantibodies against the respective antigens. However, further confirmatory testing is required for the definite determination of each specificity.

## Cytoplasmic Patterns

The classification tree for cytoplasmic patterns is also presented in Figure 1 and representative images are shown in Figure 2. Cytoplasmic patterns are defined as any staining of the HEp-2 cytoplasm, irrespective of positive or negative staining of nuclei or mitotic cells. The five major pattern subgroups fibrillar, speckled, reticular/mitochondrion-like, polar/Golgi-like, and rods and rings (RR) are considered competent-level reportable for laboratories that perform ANA IIF tests. The nomenclature is primarily based on the reactivity (staining characteristics) observed in the cytoplasm (cf. fibrillar or speckled) and the cytoplasmic structure that is recognized (cf. rods and rings). If there is a very strong correlation with the target autoantigen or the intracellular structure that is recognized, the name of the respective antigen or intracellular structure is used with addition of “-like” (cf. Golgi-like). Pattern associations with autoantigens and diseases are summarized in Table 3.

**Table 3:** **Synonyms for cytoplasmic patterns and association with specific antigens and diseases**.

		Synonyms	Antigen associations	Disease association
*Cytoplasmic patterns*
	***Fibrillar*** (AC-15,16,17)			
	Linear/actin (AC-15)	Actin-like	Actin, non-muscle myosin	MCTD, chronic active hepatitis, liver cirrhosis, myasthenia gravis, Crohn’s disease, PBC, long-term hemodialysis, rare in SARD other than MCTD
	Filamentous/microtubules (AC-16)		Vimentin, cytokeratins	Infectious or inflammatory conditions, long-term hemodialysis, alcoholic liver disease, SARD, psoriasis, healthy controls
	Segmental (AC-17)		Alpha-actinin, vinculin, tropomyosin	Myasthenia gravis, Crohn’s disease, ulcerative colitis

	***Speckled*** (AC-18–20)			
	Discrete dots (AC-18)	GW body, processing body, lysosome*	GW182, Su/Ago2, Ge-1	PBC, SARD, neurological and autoimmune conditions
	Dense fine speckled (AC-19)	Homogeneous	PL-7, PL-12, ribosomal P proteins	“anti-synthetase syndrome,” PM/DM, SLE, juvenile SLE, neuropsychiatric SLE
	Fine speckled (AC-20)	Speckled	Jo-1/histidyl-tRNA synthetase	Anti-synthetase syndrome, PM/DM, limited SSc, idiopathic pleural effusion

	***Reticular/AMA*** (AC-21)	Mitochondrion-like	PDC-E2/M2, BCOADC-E2, OGDC-E2, E1α subunit of PDC, E3BP/protein X	Common in PBC, SSc, rare in other SARD

	***Polar/Golgi-like*** (AC-22)		Giantin/macrogolgin, golgin-95/GM130, golgin-160, golgin-97, golgin-245	Rare in SjS, SLE, RA, MCTD, GPA, idiopathic cerebellar ataxia, paraneoplastic cerebellar degeneration, viral infections

	***Rods and rings*** (AC-23)		IMPDH2, others	HCV patients post-IFN/ribavirin therapy, rare in SLE, Hashimoto’s and healthy controls

*These disease associations are primarily based on the antigens recognized by antibodies that reveal this particular ANA pattern. Amber background are recommended as competent-level reporting, whereas all others (Olive green) are considered for expert-level reporting*.

*no molecular evidence to support this pattern is associated with lysosomal targets.

### Fibrillar cytoplasmic

The fibrillar cytoplasmic patterns include linear, filamentous, and segmental patterns. The fibrillar linear pattern is characterized by decorated cytoskeletal fibers, sometimes with small, discontinuous granular deposits. Target autoantigens include actin exhibiting striated actin “cables” spanning the long axis of the cells. A similar staining was reported for antibody to the heavy chain of non-muscle myosin (32). The fibrillar filamentous pattern describes filaments and fibrils spreading out from the nuclear rim, often concentrated around the nucleus and extending into the cytoplasm (Figure 2E). Typical antigens include vimentin and cytokeratins. The fibrillar segmental pattern includes enhanced decoration of short segments, periodic dense bodies, along the stress fibers. Autoantigens include alpha-actinin, vinculin, and tropomyosin.

### Speckled cytoplasmic

Within the major group of speckled cytoplasmic patterns, three minor patterns can be distinguished. Several patterns with discrete cytoplasmic dots have been described based on the number and distribution of dots in the cytoplasm. These patterns have been categorized as the discrete dots/GW body-like pattern (Figure 2F). Discrete, countable foci, known as GW bodies, are irregularly distributed throughout the cytoplasm, although they tend to be in closer proximity to the nuclear envelope (33). Immuno-gold electron microscopy has demonstrated that they range from 100 to 300 nm in diameter and are devoid of a lipid bilayer membrane. GW bodies are small in early S phase and larger during late S and G2 phases of the cell cycle. The majority of GW bodies disassemble prior to mitosis and small GW bodies reassemble in early G1. Known autoantigens within GW bodies include Su/Argonaute-2, Ge-1, and GW182. Historically, this staining pattern was thought to represent anti-lysosome antibodies, but lysosomal target(s) displaying this staining pattern have not been defined (34). Therefore, it may be that the lysosome nomenclature that appears in some publications is incorrect. Other discrete cytoplasmic dots patterns include autoantibodies staining early endosome antigen 1 (EEA1), and the cytoplasmic linker protein, CLIP 170.

The cytoplasmic dense fine speckled/homogeneous pattern appears as a cloudy, almost homogeneous, speckled pattern throughout the cytoplasm and is sometimes referred to as homogeneous cytoplasmic. Autoantibodies associated with this pattern include PL-7 or PL-12 in PM/DM, ribosomal P proteins in SLE, in particular, juvenile and neuropsychiatric, and AIH. It should be noted that this staining pattern is neither sensitive nor specific for the autoantibodies directed to these targets.

In case of the cytoplasmic fine speckled/speckled pattern, small speckles are scattered in the cytoplasm mostly with homogeneous or dense fine speckled background. Possible autoantibodies are against aminoacyl-tRNA-synthetases, mainly Jo-1 (histidyl-tRNA synthetase). The main clinical associations are PM/DM and the “anti-synthetase syndrome” (myositis, interstitial lung disease, mechanics hands, arthritis, Raynaud’s phenomenon). There are some concerns that this cytoplasmic fine speckled pattern may be a pattern association with selected HEp-2 substrates. Further work may be needed to validate putative antibody associations.

### Reticular/mitochondrion-like (AMA)

The cytoplasmic reticular/mitochondrion-like pattern is represented as a characteristic coarse granular filamentous staining extending from the nuclear envelope and tending to taper off near the outer cytoplasm and cell membrane. The main autoantigens are localized to the inner mitochondrial membrane and consist of E2 components of the 2-oxoacid dehydrogenase family of enzyme complexes (2-OACD), including pyruvate dehydrogenase complex (PDC-E2), branched chain 2-oxoacid dehydrogenase complex (BCOADC-E2), 2-oxo-glutarate dehydrogenase complex (OGDC-E2), the E1α subunit of PDC, and E3 binding protein (E3BP/protein X) (35). Anti-centromere, anti-Sp100, antinuclear envelope, and anti-GW body antibodies can be occasionally observed together with anti-mitochondria antibodies (AMA). AMA and anti-Sp100 are highly associated with primary biliary cirrhosis (PBC), and may precede the disease onset for years and even decades. The presence of anti-centromere antibodies is an indicator of current or evolving limited cutaneous SSc and, in some patients, PBC may also develop during the clinical course of the disease.

### Polar/golgi-like

This pattern is characterized by a perinuclear arrangement of coarse granules or lamellae on one pole of the cell corresponding to the stacks of the Golgi complex (Figure 2G). Known autoantigens recognized in this pattern are giantin/macrogolgin, golgin-95/GM130, golgin-160, golgin-97, and golgin-245 (36, 37).

### Rods and rings

In HEp-2 cell ANA slides from certain manufacturers (16–18), rods and rings (RR) structures present themselves in two major forms, discrete filamentous “rods” 3–10 mm in length, or annular “rings” 2–5 mm in diameter (38). Both forms are observed primarily in the cytoplasm, although generally smaller structures are regularly found in the nucleus under cellular conditions allowing for RR formation. While it has been shown that RR are not associated with any known organelles, perinuclear rods that appear to wrap around or position along the cytoplasmic side of the nuclear membrane have been observed (Figure 2H). Usually, 1–2 RR structures are observed in each cell. The major autoantigen is inosine monophosphate dehydrogenase 2 (IMPDH2) the rate-determining enzyme in the GTP biosynthetic pathway (38, 39). The overwhelming clinical association is HCV under treatment with α-interferon and ribavirin.

## Mitotic Patterns

**Figure 3:**
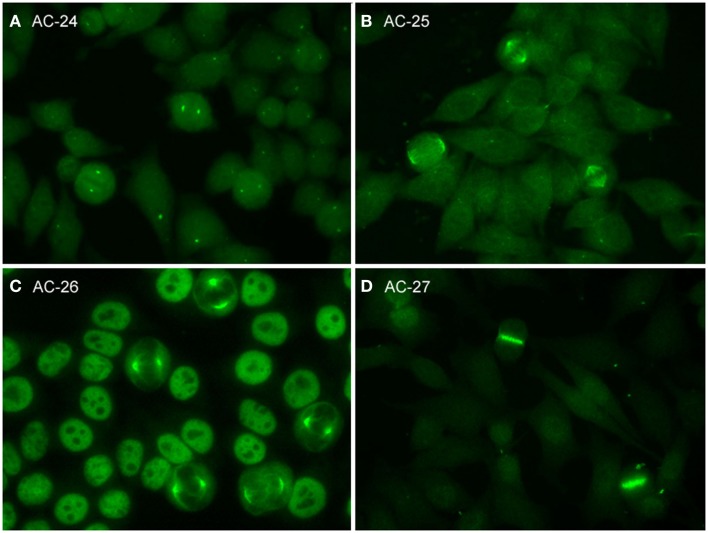
**Representative images of mitotic HEp-2 cell patterns**. **(A)** centrosome (AC-24); **(B)** spindle fibers (AC-25); **(C)** NuMA-like (AC-26); **(D)** contractile ring and intercellular bridge (AC-27).

Patterns that address cell domains strongly related to mitosis are classified as mitotic patterns (Figures 1 and 3). Some patterns that stain domains not exclusively associated with mitosis were classified as mitotic patterns if they exhibit very distinctive features during mitosis. For example, centrosomes are easily recognized as two bright spots in the mitotic cell usually aligned at opposite sides of the metaphase plate, but in the interphase cell a single less bright and less specific spot is seen in the cytoplasm. Therefore, the centrosome pattern was categorized as a mitotic pattern. The NuMA pattern is characterized as strong and characteristic staining of the pericentriolar region and the mitotic spindle, but it also has a compact speckled staining of interphase nuclei. Therefore, the NuMA pattern was classified as a mitotic pattern. Pattern associations with autoantigens and diseases are summarized in Table 4.

**Table 4:** **Synonyms for mitotic patterns and association with specific antigens and diseases**.

		Synonyms	Antigen associations	Disease association
*Mitotic patterns*
	Centrosome (AC-24)	Centrioles	Pericentrin, ninein, Cep250, Cep110, enolase	Rare in SSc, Raynaud’s phenomenon, infections (viral and mycoplasma)

	Spindle fibers (AC-25)	MSA-2	HsEg5	Rare in SjS, SLE, other SARD
	NuMA-like (AC-26)	MSA-1	Centrophilin	SjS, SLE, other

	Intercellular bridge (AC-27)	Stem body, midbody	Aurora kinase B, CENP-E, MSA-2, KIF-14, MKLP-1	Rare in SSc, Raynaud’s phenomenon, malignancy

	Mitotic chromosome coat (AC-28)	Chromosome coat protein, dividing cell antigen, mitotic chromosome autoantigen (MCA)	Modified histone H3, MCA-1	Rare in discoid lupus erythematosus, chronic lymphocytic leukemia, SjS, and polymyalgia rheumatica

*These disease associations are primarily based on the antigens recognized by antibodies that reveal this particular ANA pattern*.

### Centrosome

The pattern of antibodies against centrosome proteins imposes most often in metaphase cells as two bright spots at the spindle poles (Figure 3A). During the stages between prophase and metaphase, two neighboring bright spots can be seen with an increasing space gap as the mitotic cycle proceeds toward metaphase. In interphase cells, one single spot in most cells is visible relatively close to the nuclear envelope. Some interphase cells, usually at G2 phase, may present a pair of adjacent dots. Centrosomes are organelles located in the cytoplasm and consisting of two orthogonally arranged centrioles embedded in an amorphous mass of proteins. The term anti-centriole pattern has been used synonymously. However, it was argued that the reactivity is rarely restricted to the centrioles themselves. Therefore, it was acknowledged that the term anti-centrosome is the preferred term. The target autoantigens may be multiple components of centrosome including enolase (40), pericentrin, PCM-1, ninein, and Cep250 (41).

### Spindle fibers

Autoantibodies to spindle fibers have been reported in several studies (42–45). In metaphase cells, a bright staining of the whole spindle apparatus from the pole to the chromatin plate occurs. Fibers as well as centrosomes are stained, but not the chromosome plate (Figure 3B). No consistent staining is seen in interphase nuclei. In prophase cells, antibody binding may be first seen as staining of centrosomes. Metaphase cells present a peculiar fluorescence of the complete mitotic spindle apparatus which is also seen in the anaphase. In telophase, a staining of the edges of the intercellular bridge is observed. Some sera may occasionally stain the midbody region as well. One target autoantigen is a 130-kDa protein termed HsEg5, a kinesin-like protein involved in microtubule assembly of mitotic spindles (45).

The nuclear mitotic apparatus (NuMA) pattern, classified as a subpattern of spindle fibers, cannot be identified on one single cell but is a characteristic summary picture of many cells in different stages of the cell cycle (Figure 3C). The nucleoplasm of interphase cells depicts a compact fine speckled pattern, generally in high titer and sparing the nucleoli. Cells in metaphase and anaphase show a strong staining of spindle poles and the proximal parts of spindle fibers, also described as staining of the triangular or banana-shaped pole area. The innermost part of the centrosome remains unstained, yielding a ring-like appearance. In telophase cells, no staining of the intercellular bridge is seen, while the newly forming nuclei become diffusely stained as telophase advances. The target antigen is a 210-kDa centrophilin that is not only localized in the pericentrosomal region of mitotic cells but also present in interphase nuclear matrix (46).

### Intercellular bridge

The category intercellular bridge comprehends several distinct patterns primarily affecting structures that refer to the latest phases of mitosis, i.e., telophase and cytokinesis, localizations like the cleavage furrow, the contractile ring, the midbody region, and the stem body (Figure 3D). Most of these patterns yield no consistent staining in the interphase cells but in prophase and metaphase cells, fluorescence may be observed in the chromosomal region and be seen as fine zipper-like streaks perpendicular to the edge of the metaphase plate, representing the contractile ring. In telophase cells, the staining is usually restricted to the cleavage furrow with two bright spots at the boundary point of both daughter cells (11). In interphase, namely the S and G2 phase, cells may be stained with discrete or patchy nuclear speckles (12).

The midbody is a mitotic domain that develops during the separation of the two daughter cells in cytokinesis. It originates from the central spindle in anaphase and later midzone in telophase. It was recommended (47) that the term midbody be used only for patterns staining exclusively the midbody, i.e., the central part within the bridge. The midbody is formed of more than 150 different constituents, structural proteins, and many motor proteins (kinesins). Antibodies to kinesin 14 are characterized by an exclusive staining of the midzone and the midbody. In the center of the midbody, a bulge-like structure named the stem body can be found (48).

The target antigens recognized by these autoantibodies have not been completely identified, but aurora kinase B (49), an enzyme involved in the attachment of the mitotic spindles to centromeres, is a possible candidate. Several molecules, such as CENP-E, MSA-2, KIF-14 and MKLP-1, are also reported as midbody proteins (47).

### Mitotic chromosome coat

Cells in pro- and metaphase show a fine granular staining of the chromosomal surface. The inner part of condensed chromosomes remains unstained as well as the nucleoplasm of interphase cells (47, 50, 51). However, since the autoantigens have not been reported, the true prevalence of the autoantibody remains to be determined. The inclusion of this pattern may stimulate further investigations.

## Discussion

The first Brazilian ANA consensus established a decision tree with the focus on the morphological criteria for pattern recognition and aimed to provide guidance for reading slides (8). The second Brazilian ANA consensus extended the decision tree and added some considerations for mixed patterns as well as the relevant clinical associations for each pattern (9). The third ANA consensus included new patterns and some recommendations for quality controls (10). It was also proposed to exclude the use of transfected HEp-2 cells (i.e., HEp-2000 substrate, ImmunoConcepts Inc., Sacramento, CA, USA) for general pattern definition (10). The more recent 2013 consensus added new patterns, such as RR and CENP-F, and discussed the impact of automated ANA screening (11). Building on the Brazilian experience, it was clear that the first ICAP needed to limit its scope and plan to advance this initial focus by establishing and publishing the consensus of the assembled experts from wide geographic jurisdictions. Accordingly, the primary goal of the first ICAP was to establish a platform to promote and eventually reach a high level of consensus. By proposing the preliminary nomenclature, classification tree and a set of images for each autoantibody staining pattern on the internet, we hope to stimulate discussion that will lead to subsequent ICAP deliberations. Thus, the next rounds of ICAP will successively lead to improvements in the nomenclature and pattern identification based on the feedback, commentaries or criticism from the international community.

Due to the nature of the consensus-building process, it is clearly acknowledged that the first ICAP is incomplete and imperfect in several aspects. One deficiency may be the lack of attention to composite patterns and its distinction from mixed patterns. For example, anti-topoisomerase I/Scl-70 often is associated with a composite pattern but at this stage it has not been incorporated in the classification tree for nuclear patterns. As in any other ANA pattern, the autoantibody specificity should be confirmed in additional specific testing. Yet, this first ICAP included another composite pattern, namely the nuclear Dense Fine Speckled pattern (AC-2), which has been strongly correlated to the presence of autoantibody to DFS70 and, importantly, is seen in very low frequency in SjS, SSc, and SLE (23, 24). Whether this pattern can also be used for exclusion of other SARD remains to be determined through systematic study of a wider range of diseases. With respect to disease associations reported in Tables 2–4, it should be noted that the ANA assay is primarily a screening assay. Positive results of ANA screening tests should be titrated to end-point and further investigated with antigen-specific assays. Thus, the disease associations listed in Tables 2–4 are primarily based on the antigens recognized by their cognate autoantibodies that reveal each particular ANA pattern; the disease associations are not meant for inclusion in the test result in the report to clinicians. It is acknowledged that the evidence for these clinical associations varies widely ranging from single published reports to multiple studies and readers are advised to consider each with this reservation in mind.

Future attention should be devoted to clinically relevant mixed patterns. A mixed pattern is defined when a patient has multiple autoantibodies that recognize more than one simple pattern. For example, in PBC, it is not uncommon to observe sera with autoantibodies to both centromere and mitochondria or both multiple nuclear dots and mitochondria. Since the centromere pattern has discrete nuclear dots in the interphase and mitotic cells, while the mitochondria pattern is in the cytoplasmic compartment, this mixed pattern can be readily identified. Other mixed patterns, however, can be more difficult to discriminate accurately. This is particularly true when the titers of two autoantibodies are relatively similar or when their cognate antigens are in the same cell compartment.

A second obvious omission is the failure to cover all known patterns or even all patterns that are already included in the Brazilian ANA consensus and in other similar initiatives (12). One may consider that it should be simple to adapt all known patterns described previously. However, the general focus of the ICAP is to include patterns that are more clinically relevant and obtain consensual approval of all participating experts. Rare patterns are excluded at this time; for example, cell cycle patterns including SG2NA/striatin (52), cyclin B1 (53), heterochromatin protein 1-beta (54), and M-phase phosphoprotein MPP1 (55), and EEA1 and other endosomal antigens (34, 56). It is acknowledged that there may be potential biases and not all patterns can be equally well justified for their inclusion/exclusion in this first edition of the ICAP. It is expected that a progressive maturation process occurs along the successive ICAP editions with a broad participation of the international community.

It also became clear that the CDC/IUIS ANA Reference Standards require reevaluation using contemporary ANA IIF and related technologies. In particular, it is important to clarify anti-Ro60 versus anti-Ro52 staining patterns, an issue that was not explicitly discussed in relation to the CDC/IUIS ANA standards. The CDC/IUIS ANA standard #07 reference serum for human antibodies to SS-A/Ro was established and released in May 1983 (4). The distinction of Ro52 and Ro60 was not reported until 1988 (57). At this time, there is no CDC/IUIS ANA reference standard for Ro60 alone or Ro52 alone. Other investigators have analyzed many of the CDC/IUIS ANA standards for reactivity to Ro60 and Ro52 and showed that (1) CDC/IUIS ANA standard #07 has strong reactivity to Ro60 and weak reactivity to Ro52, (2) CDC/IUIS ANA standard #02 (anti-SS-B/La) and #03 (Speckled) also have strong anti-Ro60 and weak anti-Ro52 reactivity, and (3) CDC/IUIS ANA standard #10 (anti-Jo-1) had strong additional reactivity to Ro52 (58). The current interpretation is that the subcellular localization of the majority of subcellular Ro60 and Ro52 are likely different. Anti-Ro60 shows a typical nuclear fine speckled pattern (AC-4), while anti-Ro52 does not show nuclear staining (59). In fact, many monospecific anti-Ro52 do not have an identifiable staining pattern. There have also been concerns that not all anti-Ro antibodies give nuclear staining and that may be explained in part from the autoantibody titer, presence of other autoantibodies that may interfere, and/or the use of certain brand of ANA slide substrates that are known not to detect anti-Ro as nuclear pattern.

In summary, in this first ICAP consensus, we present a classification tree for ANA with alpha-numeric AC code for each pattern. The classification includes not only nuclear patterns but also cytoplasmic and mitotic patterns that are all considered to be ANA positive. This is an important point deviating from certain norms in ANA reporting in some clinical immunology laboratories that only “clinically relevant” ANA patterns are reported, while other “less important” patterns are ignored. We argue that all known patterns should be reported and clinical relevance acknowledged accordingly. The main concern with selective reporting, as it is practiced currently in some laboratories, is that it promotes hiding information and that potentially can be more harmful. Within the classification tree developed here, a distinction is made between competent-level and expert-level patterns based on clinical relevance and/or easiness of recognition. The goal is to promote a stepwise integration of ANA reporting to a higher level with continuing training. Future ICAP editions are expected to further delineate this consensus, resulting in widely embraced standardization of ANA testing and reporting.

## Author Contributions

EC and LA conceived the ICAP initiative. LA organized and coordinated the ICAP meeting and invited participants. All authors, except LA, WC, and MF participated as discussion leaders during the ICAP meeting. EC, OC, PF, IT, MH, CM, and LA contributed images for initial selection to be included in this manuscript and the ANApatterns.org website. Everyone except KC voted on the images representing each ANA patterns. JD, CM, EC, MH, and TM wrote sections of the first manuscript draft. WC and EC organized and designed the www.ANApatterns.org website. All authors participated in reviewing and editing of the final manuscript.

## Conflict of Interest Statement

The authors declare that the research was conducted in the absence of any commercial or financial relationships that could be construed as a potential conflict of interest.

## Supplementary Material

The Supplementary Material for this article can be found online at http://journal.frontiersin.org/article/10.3389/fimmu.2015.00412

## Abbreviations

AC, anti-cell; AIH, autoimmune hepatitis; ALD, alcoholic liver disease; AMA, anti-mitochondrial antibodies; ANA, antinuclear antibody; CDC, Centers for Disease Control and Prevention; CENP-F, centromere-associated protein F; CLD, chronic liver diseases; DFS, dense fine speckled; DM, dermatomyositis; EBV, Epstein–Barr virus; HCV, hepatitis C virus; HIV, human immunodeficiency virus; IIF, indirect immunofluorescence; IMPDH2, inosine monophosphate dehydrogenase 2; IUIS, International Union of Immunological Societies; MCTD, mixed connective tissue disease; NE, nuclear envelope; NHS, normal human serum; NOR, nucleolar organizer regions; PBC, primary biliary cirrhosis; PCNA, proliferating cell nuclear antigen; PM, polymyositis; RNP, ribonucleoprotein; RR, rods and rings; SARD, systemic autoimmune rheumatic diseases; SjS, Sjögren’s syndrome; SLE, systemic lupus erythematosus; SSc, systemic sclerosis.
