# Supplementary material for: Report of the First International Consensus on Standardized Nomenclature of Antinuclear Antibody HEp-2 Cell Patterns 2014–2015
Source: Front Immunol. 2015 Aug 20;6:412. doi: 10.3389/fimmu.2015.00412 (PMC4542633; doi:10.3389/fimmu.2015.00412)
Supplement: Supplementary file 1 [file Table_1.PDF]

Supplemental Table 1: Participants at the first International Consensus on Antinuclear Antibody HEp-2 Cell Patterns (ICAP) standardization session during the 12th International Workshop on Autoantibodies and Autoimmunity meeting held in Sao Paulo, Brazil, on August 28, 2014.

Name	Country
Adriana Roy	Argentina
Adriana Cristina Carvalho Degenhardt	Brazil
Ajax Atta	Brazil
Alejandra Ginaca	Argentina
Alice Friedenberg	Brazil
Ana Maria Sales Bueno Teixeira	Brazil
Ana Patricia do Nascimento	Brazil
Ana Paula Carvalho	Brazil
Andre Rocha	Brazil
Andréa Colado Simão	Brazil
Anna Maria Barancelli	Brazil
Antonio Cardinalli	Argentina
Camilo Zurita	Ecuador
Carlos Nuñez	Mexico
Carlos David Araujo Bichara	Brazil
Carol Buchner	Brazil
Catlina Perez	Brazil
Christian Blas La Rosa Fabian	Peru
Cleandro Pires de Albuquerque	Brazil
Cynthia Liedstrand	USA
Daman Langguth	Australia
Deborah Yasmin de Sousa	Brazil
Donglai Ma	Canada
Ed Bass	Brazil
Eric Hoy	USA
Fabiano Almeida Brito	Brazil
Fabio Montera	Brazil
Fernanda Bull Flumian	Brazil
Fernando Antunez Borowy	Uruguay
Flávio Oliveira Paiva	Brazil
Gladys Orfus	Argentina
Isabel Abreu	Portugal
Jackie Mackay	Brazil
Jose Fernando de Souza	Brazil
Josiane Silveira Santanna	Brazil
Lakshmanan Suresh	USA
Luciana Carolina Eidenson	Argentina
Luis Broggi	Brazil
Luís Gonzaga Moura Xavier	Brazil
Marcela Tafur	Colombia
Margarete Vendramini	Brazil
Maria Ester Mendonça Salvador	Brazil
Maria Fernanda Severini	Brazil
María Laura Strada Agodino	Argentina
Maria Lucia Alvares de Azevedo Bahr	Brazil
Maria Luiza Brito de Sousa Atta	Brazil
Maria Teresa Maluf Chamma	Brazil
Marta Costa	Argentina
Michael Mahler	USA
Muriel Berti	Brazil
Patricia Carabajal	Argentina
Qingyong Xu	Canada
Ricardo Amaro Noleto Araujo	Brazil
Roberta Silveira	Brazil
Roger Walker	Brazil
Rogerio Maria	Brazil
Rosemeire Midori Yamada	Brazil
Sandra Gofinet Pasoto	Brazil
Silvana Zanaga	Brazil
Silvia González	Brazil
Suzane Pretti Figueiredo Neves	Brazil
Vanessa Alves da Silva	Brazil
Victor Bercot	Brazil
Werner Klotz	Austria
Wilson Melo Cruvinel	Brazil
Wilton Santos	Brazil
