# Supplementary material for: Report of the First International Consensus on Standardized Nomenclature of Antinuclear Antibody HEp-2 Cell Patterns 2014–2015
Source: Front Immunol. 2015 Aug 20;6:412. doi: 10.3389/fimmu.2015.00412 (PMC4542633; doi:10.3389/fimmu.2015.00412)
Supplement: Supplementary file 2 [file Table_2.PDF]

Supplemental Table 2: Representatives from fifteen countries at the first ICAP standardization session during the 12th International Workshop on Autoantibodies and Autoimmunity meeting held in Sao Paulo, Brazil, on August 28, 2014.*

Country	Number of participants
Argentina	9
Australia	1
Austria	2
Brazil	47
Canada	3
Colombia	1
Ecuador	1
Germany	1
Japan	2
Mexico	2
Netherland	1
Peru	1
Portugal	1
Uruguay	1
USA	5
total	78

*Includes work group leaders and meeting attendants.
